# A digital audio workstation approach for matching the sound quality of speech and music for single-sided deaf patients fit with cochlear implants

**DOI:** 10.3389/fnins.2026.1798569

**Published:** 2026-04-09

**Authors:** Stephen R. Dennison, Katelyn A. Berg, Sarah C. Natale, Beatriz de Diego-Lázaro, David Carabias-Galindo, Alberto Acebes-de-Pablo, Joshua S. Stohl, Michael F. Dorman

**Affiliations:** 1 MED-EL Corp., Durham, NC, United States; 2 Washington University, St. Louis, MO, United States; 3 Arizona State University, Tempe, AZ, United States; 4 Universidad de Valladolid, Segovia, Spain

**Keywords:** bimodal, cochlear implants, digital audio workstation, music, single-sided deaf, speech quality

## Abstract

**Introduction:**

Cochlear implant (CI) patients who are single-sided deaf can match the sound quality of speech presented to their CI ear and speech presented to their normal hearing ear. Previous work using this patient population has generated acoustic approximations of CI sound quality for speech, achieving high similarity ratings through interactive manipulation of sound parameters such as filtering, pitch shifting, and spectral smearing. The present study aimed to extend this approach to music.

**Methods:**

A digital audio workstation (DAW) methodology was developed for generating sound quality matches to both speech and music in 11 adults with unilateral MED-EL CIs and contralateral acoustic hearing. Participants compared the sound quality created by acoustically manipulated signals presented to their better hearing ear with the sound quality of unprocessed signals presented to the CI ear. The similarity of the two signals was rated on a scale of 1 to 10 with 10 indicating a perfect match.

**Results:**

On average, speech matches achieved higher similarity ratings (9.3) than music matches (6.7). Speech matches were typically achieved using bandpass filtering, pitch shifts, and distortion. Similarity ratings for speech using the digital audio workstation (9.3) were not different from those (8.7) using the custom, speech-specific software of previous studies. Music matches frequently required additional manipulations, including frequency equalization and modulation. The specific manipulations required varied across participants, and several individuals could not complete music matches despite extensive attempts.

**Discussion:**

These findings suggest that music introduces perceptual dimensions not fully addressed by speech-based matching procedures. The DAW methodology provides an accessible framework for investigating CI sound quality and may guide future efforts to characterize and optimize sound quality for signals beyond speech.

## 1 Introduction

A fundamental question in the field of cochlear implants (CIs) is “what does a CI sound like?” Traditionally, post-lingually deafened CI patients have relied solely on memories of acoustic hearing to evaluate CI sound quality, limiting the precision of such assessments. However, the expansion of CI candidacy criteria to include patients with single-sided deafness (SSD) (Park et al., 2022) has created a novel research opportunity. These SSD patients, who retain normal or near-normal hearing in the contralateral ear, can provide direct, real-time comparisons between acoustic hearing and electrical stimulation (Dorman et al., 2020). This unique population has enabled researchers to directly measure CI sound quality in ways that were previously not possible.

Beginning in 2017, Dorman and colleagues developed a systematic method for quantifying CI sound quality with SSD patients who have CIs (Dorman et al., 2017). These investigations demonstrated that traditional vocoder simulations poorly matched CI sound quality, with mean similarity scores of only 1.7 and 2.9 out of 10 for noise and sine vocoders, respectively, suggesting that simulations created with natural speech signals would be more useful. Subsequent methodological refinements expanded the acoustic manipulation parameters for natural speech to include modifications of voice pitch, intonation, formant frequencies and bandwidths, temporal and spectral smearing, and the addition of metallic or flanged qualities (Dorman et al., 2020). Through an iterative, interactive process, patients adjusted these parameters until they reported achieving close perceptual matches to their CI sound quality. On a 10-point analog scale, with 10 indicating a complete match to CI sound quality, the mean match was 8.7, with some patients achieving scores of 10.

The characteristics of these matches varied substantially with electrode length. Patients with shorter electrodes ( ≤ 18.5 mm) frequently reported “Mickey Mouse” voice quality characterized by upshifts in fundamental frequency (10–80 Hz) and formant frequencies (100–320 Hz), consistent with incomplete cochlear coverage (Dorman et al., 2019). Patients with longer electrodes (28 mm) showed two distinct patterns: some required a narrow bandwidth (0.6–1.6 kHz) with heavy smearing, while others preferred a wider bandwidth (2.9–8 kHz) with minimal smearing (Dorman et al., 2024). The rarity of 10 out of 10 similarity matches in this study indicated that available acoustic manipulations in the current tools may not fully capture all dimensions of CI sound quality, that certain effects of electrical stimulation cannot be approximated through acoustic signals to a normal-hearing ear, or both. These findings revealed that electrode array length fundamentally influenced the perceptual characteristics of CI sound quality. A separate questionnaire study found that while conventional CI users initially described their CI as “Computer-like,” “Treble-y,” “Metallic,” or “Mickey Mouse-like,” about two-thirds eventually described sound quality as “Clear” after years of use, suggesting significant neural adaptation to the CI (Dorman et al., 2025).

Tóth et al. (2023) demonstrated another application of the SSD testing paradigm for characterizing CI sound perception. In their study, 12 SSD CI users compared the pitch of acoustic tones presented to their NH ear with the pitch perceived from individual CI electrodes, enabling the creation of customized frequency allocation tables that improved subjective hearing quality. Most recently, Kopsch et al. (2025) were able to generate very high average similarity scores of 9.7 out of 10 using manipulations such as comb filtering or low pass filtering with very few additional optimizations. However, the acoustic manipulations for a single stimulus led to significantly lower similarity scores (e.g. 8.4 or 8.9 out of 10) when applied to novel speech stimuli, suggesting that manipulations may not generalize to more complicated or unfamiliar stimuli.

No studies have reported attempts to extend the SSD matching paradigm to music. This represents a critical gap in the literature, particularly given increasing interest in enhancing musical experiences for CI users (Berg et al., 2025b) and well-documented differences between speech and music perception with CIs (Bleckly et al., 2024). The extent to which the matching paradigm used by Dorman et al. (2020) to characterize the sound quality of speech can be used to match the sound quality of music is unknown.

The current study aimed to address this issue by establishing a methodology for generating sound quality matches to both speech and musical samples using a digital audio workstation (DAW). We hypothesized that, due to the greater temporal and spectral complexity of music relative to speech, similarity ratings for music would be lower than those achieved for speech, and that more extensive acoustic manipulations would be required to approximate musical sound quality. We also aimed to compare the DAW approach to previous methodology used for just speech samples. This exploratory investigation characterized the perceptual differences between speech and music as experienced through CIs, providing a foundation for future research aimed at optimizing music perception for CI users.

## 2 Materials and methods

### 2.1 Participants

**Figure 1:**
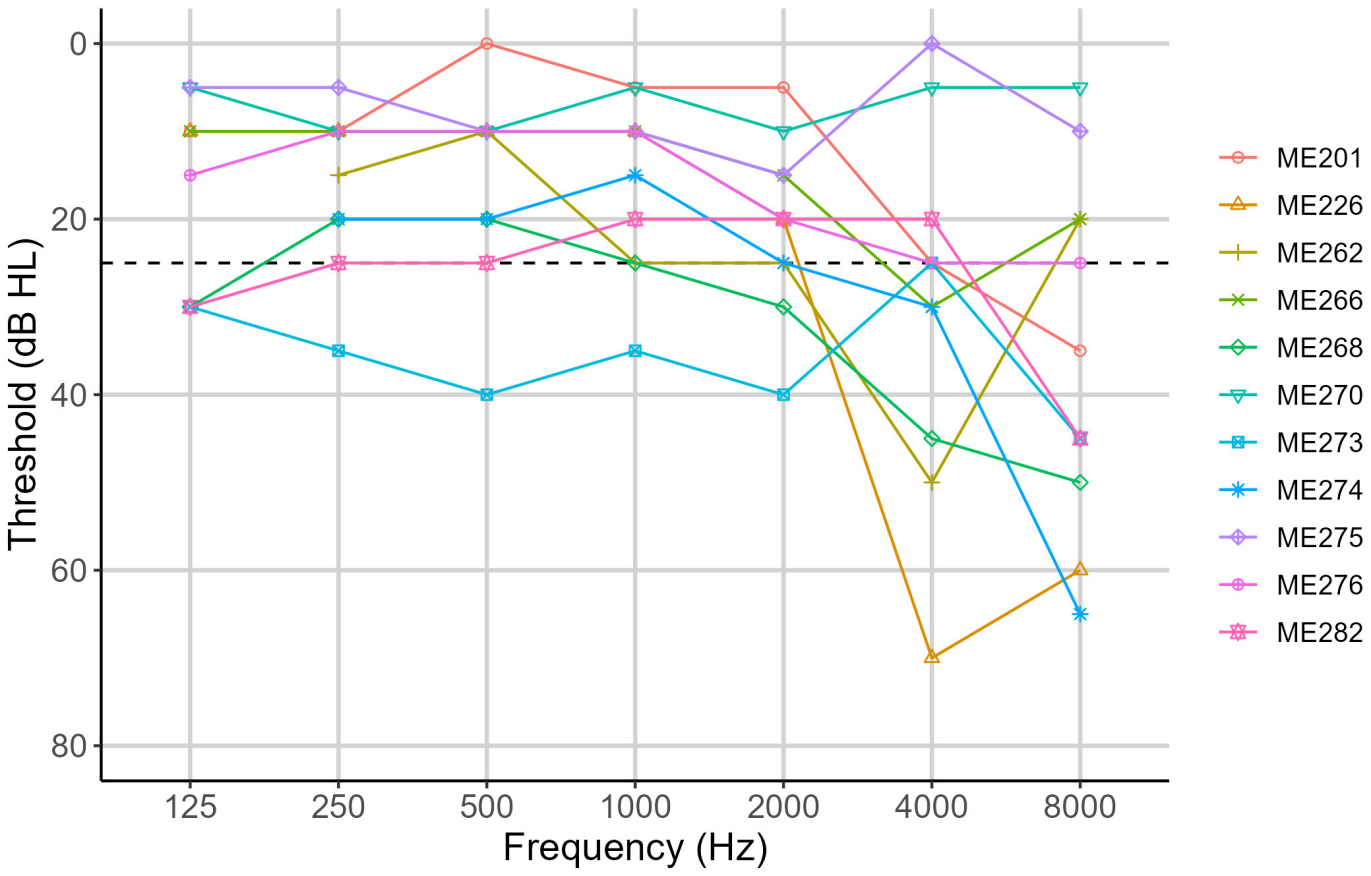
Unaided hearing levels in the non-implanted ear. The black dashed line represents 25 dB. Lines above 25 dB indicate normal hearing. Lines below 25 dB indicate hearing loss.

Eleven participants were recruited for this experiment, as summarized in Table 1. The inclusion criteria were that all listeners were required to have a MED-EL (Innsbruck, Austria) CI in one ear and residual hearing in the other ear. Three of the participants wore a hearing aid (HA) in their non-implanted ear. There were no restrictions on electrode array; six participants had MED-EL 31.5-mm FLEXsoft arrays, one had a 31.5-mm Standard array, and four had 28-mm FLEX28 arrays. All participants used the FS4 sound coding strategy in their everyday life, except one participant (ME282), who used FS4-p. Both sound coding strategies aim to provide a combination of envelope and temporal fine structure information. Audiograms for the ear contralateral to the CI are shown for each participant in Figure 1. Table 1 also shows whether participants self-reported as having musical experience.

**Table 1:** Participant information.

ID	Sex	Age	Hearing type	Etiology	Duration of CI use (months)	Self-reported musical experience
ME201	M	75	SSD, CI right	Sudden hearing loss	119	Non-musician
ME226	M	57	SSD, CI left	Labyrinithitis	56	Musician
ME262	M	62	Bimodal, CI right	Sudden hearing loss	31	Musician
ME266	M	52	SSD, CI left	Acoustic neuroma (removed at time of CI surgery)	24	Musician
ME268	M	66	Bimodal, CI left	Sudden hearing loss	33	Non-musician
ME270	F	41	SSD, CI left	Sudden hearing loss	6	Musician
ME273	M	67	Bimodal, CI left	Airplane pressurization issue, workplace injury in 2019	58	Musician
ME274	F	59	SSD, CI left	As a result of complications from a stapedectomy	24	Non-musician
ME275	F	28	SSD, CI left	Not sure, viral infection	6	Musician
ME276	M	57	SSD, CI left	Sudden sensori-neural hearing loss, sickness	22	Musician
ME282	F	59	SSD, CI left	Meniere's disease	28	Non-musician

SSD, Single side deafness; CI, Cochlear Implant.

Eight of the participants traveled to the North American Research Laboratory in Durham, North Carolina for testing. Three of the participants traveled to Vanderbilt University Medical Center (VUMC) for testing. All participants provided consent under Western Institutional Review Board (now WGC IRB) protocol #20100066, while the three participants tested at Vanderbilt provided additional consent under VUMC IRB #230093.

### 2.2 Test materials

Multiple speech and music samples were used. Speech samples included one sentence spoken by a male talker, “The sun is finally shining”, from the City University of New York sentence corpus (Boothroyd et al., 1985) and was used previously in Dorman et al. (2022). Other sentences were spoken by a female talker: “The boy enjoyed dancing with his dog” and “I wish she would convince herself she could fly” (Spahr et al., 2012). Musical samples included a piano melody played in multiple registers and familiar music chosen by each participant (see Table 2). A simple, monophonic piano melody with antecedent and consequent musical meaning was created in a central register (similar to that of the human voice) and of short duration (“01EstMelALES”). The melody was recorded with GarageBand (Apple Inc., Cupertino, CA) software, ensuring that the sound was as close as possible to a real piano (de Diego-Lazaro et al., 2026). This melody was transposed to different registers of the piano. For speech, signal durations varied based on the sentence content, while for musical signals, approximately 30 s of each song was selected.

**Table 2:** Musical samples presented to each participant.

Participant	Musical samples
ME201	-Country Roads by John Denver -Postcard to Paris by John Denver -Perfect Symphony by Ed Sheeran and Andrea Bocelli
ME226	-Hello Goodbye by The Beatles -Sympathy for the Devil by The Rolling Stones
ME262	-Mysterious Ways by U2
ME266	-01EstMelALES (Piano Melody)
ME268	-Bohemian Rhapsody by Queen -Hey Jude by The Beatles -Over the Rainbow by Judy Garland
ME270	-01EstMelALES (piano melody) -Chromatic scale on piano
ME273	-01EstMelALES (piano melody)
ME274	-N/A
ME275	-Music for a Sushi Restaurant by Harry Styles
ME276	-Bob Caygeon by The Tragically Hip
ME282	-Don't Stop by Fleetwood Mac -Uncle John's Band by Grateful Dead -Thriller by Michael Jackson

### 2.3 Apparatus

**Figure 2:**
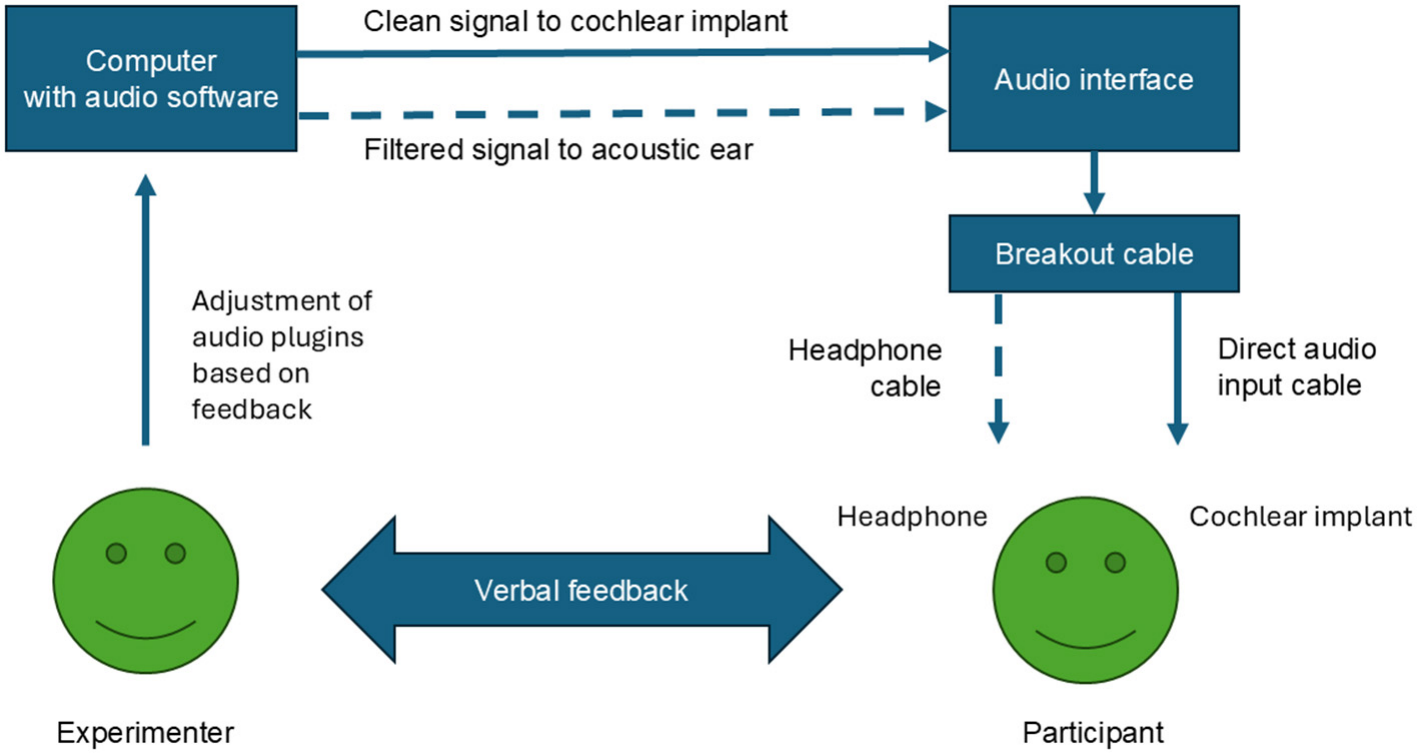
Schematic of experimental setup.

Testing was conducted in a quiet laboratory room with no sound treatment. Listeners wore a SONNET 2 processor programmed with their clinical map, or a conversion of that map for the SONNET 2. Stimuli were presented using the DAW REAPER (Cockos Incorporated, Rosendale, NY). REAPER allows the user to select stimuli and the channel through which signals are presented (acoustic hearing vs. implanted ear) quickly and easily. It also allows for on-the-fly modifications to the filters applied to stimuli through its plug-ins following participant feedback, without having to wait for re-rendering of signals. Sound was presented via a breakout cable that separated left and right channels, with one channel going to a Beyerdynamic DT 252 single-ear headphone (Heilbronn, Germany) on the non-implanted ear, and one channel going to the CI via a direct audio input (DAI) cable, as schematically represented in Figure 2. All stimuli were presented with a Windows laptop (Microsoft, Redmond, WA, USA). The original audio samples were either mono or mixed down to mono and were processed separately in the left and right channels before presentation. The unmodified audio signals were presented by routing the channel with the unprocessed, original audio to just the CI. The other channel was used to play filtered stimuli to the acoustic ear. Filters were applied non-destructively in real time based on the participant's feedback (see Figure 2). We applied Virtual Studio Technology (VST), JesusSonic (JS), or custom REAPER plugins. The names of plugins and a description of their effects are available in Supplementary Table 1.

### 2.4 Procedure

Prior to testing, the loudness in the CI and acoustic hearing ears was matched using the digital faders on the REAPER virtual mixing console. The gain in the CI ear was brought to the loudest possible level for the signal without clipping (0 dB full scale). The acoustic hearing ear was then adjusted until the participant reported that the loudness was similar across ears. The participants were then shown a list of quality descriptors used by Dorman et al. (2025) for speech samples, see Supplementary Table 2 for the list of words. The participants could select any or none of the words to help guide which plugins would be applied. From this point, the general procedure was to loop a sample of audio for the participant. The first audio sample was always “The sun is finally shining.” Following a match to this sentence, depending on the time available, either more sentences were attempted or matching to musical samples began. No training or familiarization procedure was provided prior to the first match.

The stimulus was presented sequentially by the experimenter to the CI ear (original stimulus), then to the acoustic ear (with filtering applied), then back to the CI ear (original stimulus), with this process repeating as many times as necessary to achieve a match. The experimenter applied plugins to the signal presented to the non-implanted ear and asked the participant if the sound effect matched what they heard in the CI ear. The parameters of the plugin were adjusted so that the participant could experience the range of possible manipulations for that plugin. If the plugin did not match the sound quality in the CI ear, it was not used again. If a plugin seemed to match the sound quality in the CI ear, it was kept in the signal chain and the process continued. For example, if the subject reported “muffled,” a low pass filter could be applied. If the sound was not muffled enough, the low pass filter cutoff frequency could be lowered until the participant reported that it matched the amount of muffling. With the addition of new plugins, previously used plugins still in the signal chain were sometimes adjusted to reach a closer sound quality.

Sound quality matches to speech were also made using the procedure described in Dorman et al. (2022). Similarly to the DAW approach, the participant was asked what needed to be changed to make the signal in the typical-hearing ear similar to the CI ear. For this approach, only the sentence “The sun is finally shining” was used. Manipulations were applied to signals which were resynthesized with the applied effects. The process continued until either the match was ‘very close' or there were no more parameters to adjust. The participant was then asked for a similarity rating on a 10-point scale. Testing took approximately 30 minutes.

Matches to an individual stimulus could take as little as 15 minutes or as long as an hour, depending on the complexity of the match and the number of plug-in parameters that were adjusted. Some participants were tested for one session while others were tested in multiple sessions, depending on the availability of the participant and the amount of interaction needed to achieve their matches. We emphasize that our rating scale, from 1 to 10, does not represent subjectively “good” sound quality, but rather how closely the acoustically manipulated signal matches the sound quality perceived through the CI.

## 3 Results

All matches, in addition to the clean, unprocessed stimulus samples for the non-copyrighted musical samples are available at a public repository (Dennison, 2026).

**Figure 3:**
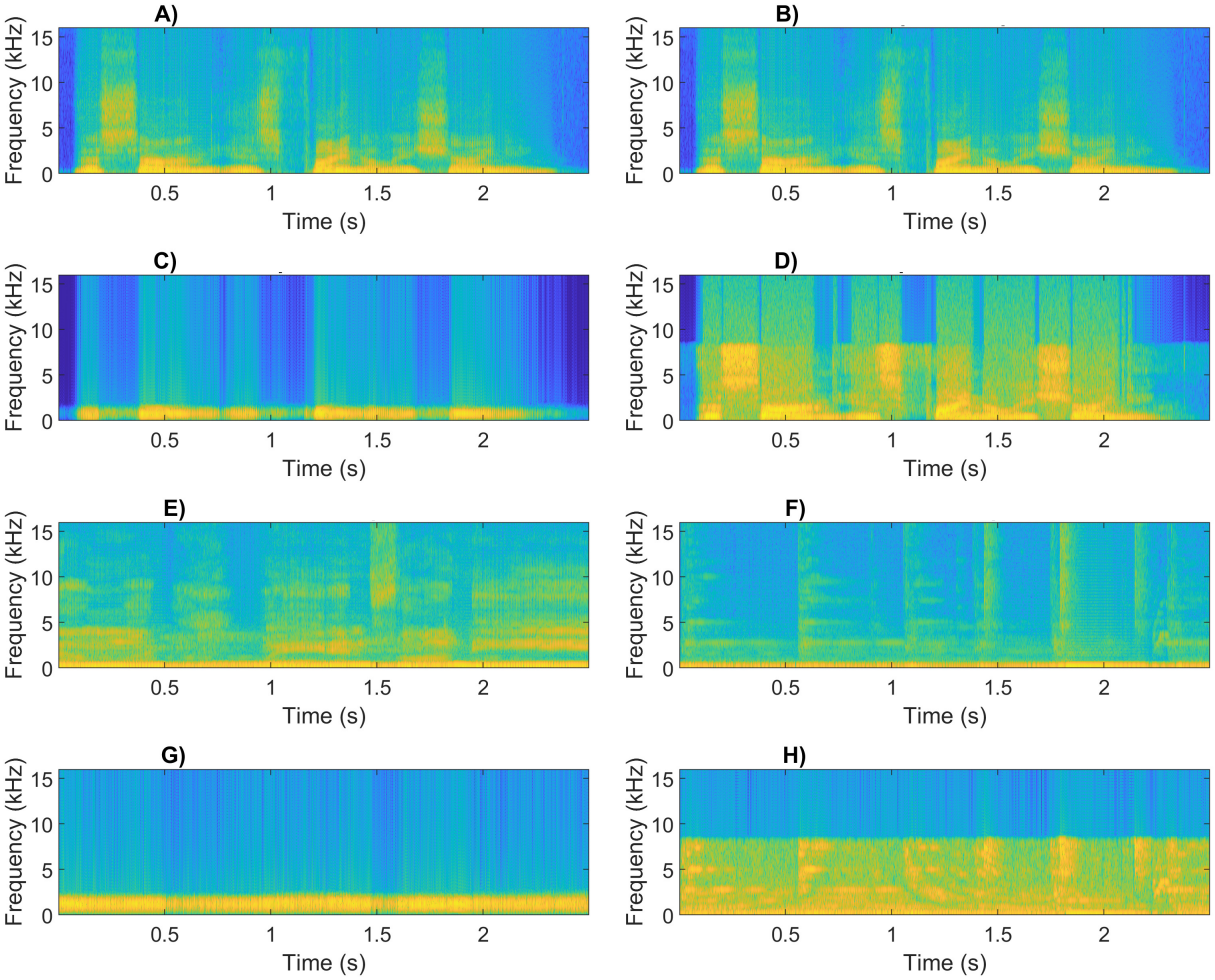
Spectrograms for ME201 and ME226 showing relatively few manipulations (ME201) vs. relatively large number of manipulations (ME226). Descriptions of the plugins applied are available in the Supplementary material. **(A)** ME201 speech unprocessed. **(B)** ME276 speech unprocessed. **(C)** ME201 speech match: 9.5/10. **(D)** ME276 speech match: 8.5/10. **(E)** ME201 music unprocessed. **(F)** ME276 music unprocessed. **(G)** ME201 music match: 9.5/10. **(H)** ME276 music match: 8.0/10.

Example spectrograms of unprocessed and matched signals are shown in Figure 3. Spectrograms were created an FFT size of 1,024 points, a Hamming window of 128 samples, and 64 samples of overlap. The audio samples for analysis were generated at a sample frequency of 44.1 kHz. A detailed description of matches to speech and music for each listener can be found in Supplementary Table 3.

**Figure 4:**
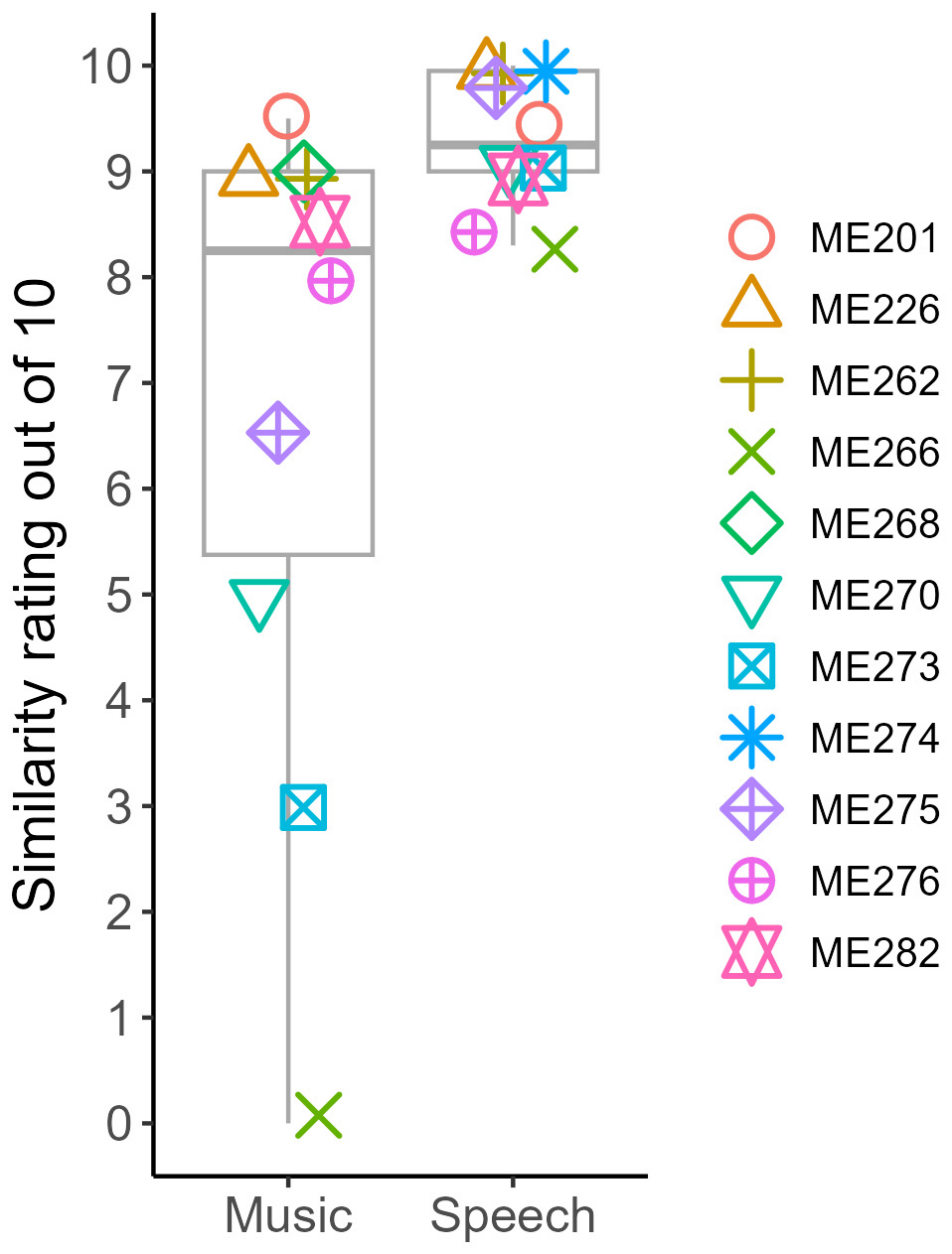
Similarity match scores for each participant.

The match scores for the signals with the closest match to CI sound quality for each participant are shown in Figure 4 and Table 3. Ten participants, all except ME268, completed speech matches. The mean similarity match score for speech signals was 9.31 out of 10 (median: 9.25 out of 10, range: 8.3 to 10 out of 10). Ten participants, not including ME274, completed music matches. The mean match score for music signals was 6.75 out of 10 (median: 8.25 out of 10, range: 0 to 9.5 out of 10). A paired-sample *t*-test for the nine participants who completed both speech and music matches revealed a significant difference between the two scores, *t* (8) = 2.83, *p* = 0.022, mean difference 2.73, 95% CI: (0.51, 4.96).

**Table 3:** Highest similarity match scores and number of plugins used for each participant.

ID	Config	Speech match	Number of plugins used	Music match	Number of plugins used
ME201	SSD	9.5	2	9.5	2
ME226	SSD	10	2	9	2
ME262	Bimodal	10	10	9	16
ME266	SSD	8.3	10	0	N/A
ME268	Bimodal	Did not test	N/A	9	6
ME270	SSD	9	4	5	12
ME273	Bimodal	9	6	3	7
ME274	SSD	10	2	Did not test	N/A
ME275	SSD	9.8	1	6.5	2
ME276	SSD	8.5	10	8	7
ME282	SSD	9	3	8.5	4

If two matches were achieved and used different numbers of plugins, the smaller number of plugins used are reported here. SSD, Single-sided deafness.

**Figure 5:**
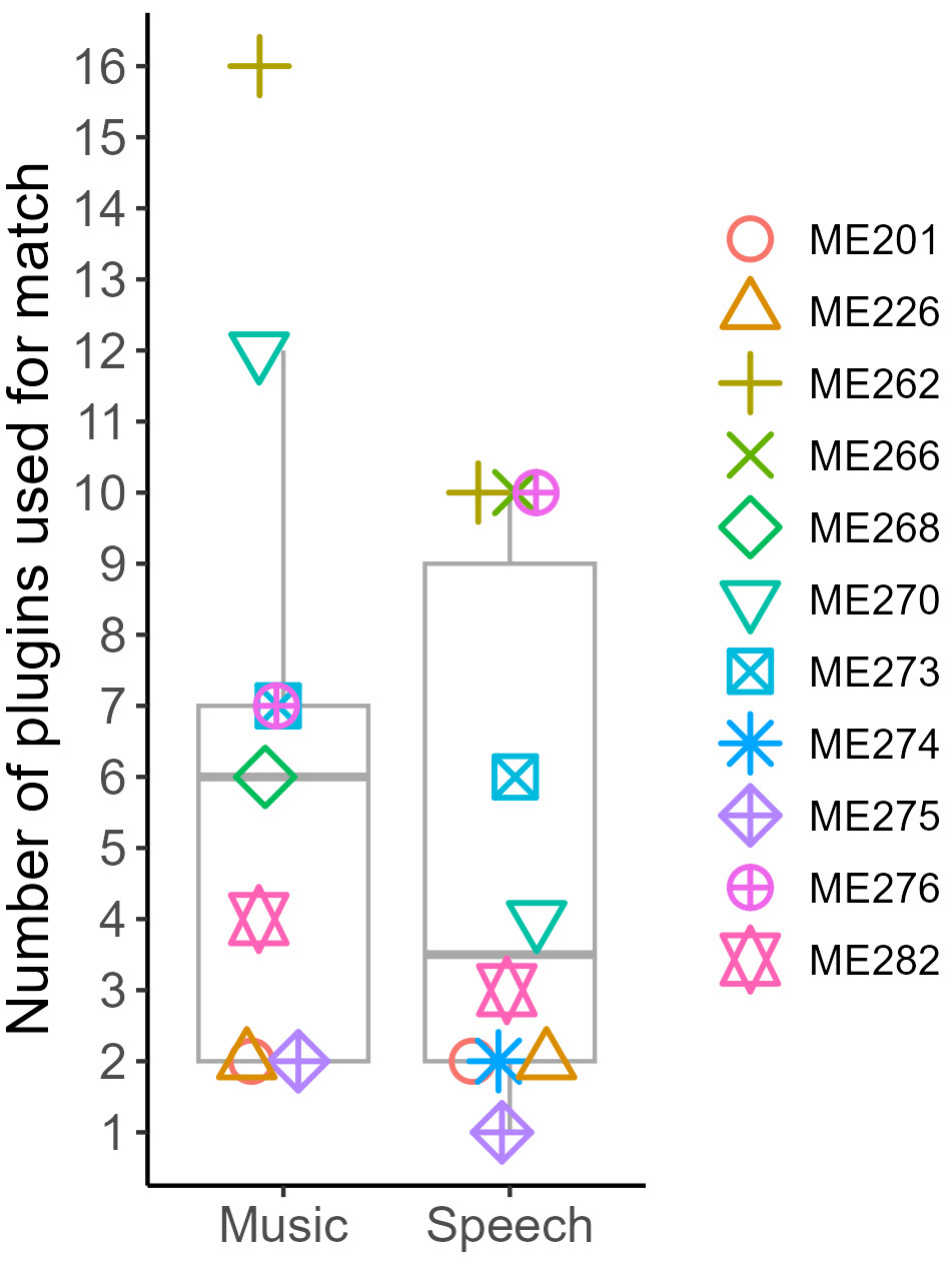
Number of plugins used for highest similarity match.

The number of plugins used for the highest similarity match for each participant is shown in Figure 5 and Table 3. We excluded the ME266 music match because of the 0 out of 10 similarity rating. On average, 5 plugins were needed to generate speech matches (median: 3.5 plugins, range: 1 to 10), and 6.4 plugins were needed to generate music matches (median: 6 plugins, range: 2 to 16). A paired-sample *t*-test for the eight participants who completed both music and speech matches (not including ME266, ME268, and ME274) revealed no significant difference between the mean scores, *t* (7) = −1.4, *p* = 0.20, mean difference −1.75, 95% CI: (−4.71, 1.21). Note that the differences in means of each group and the mean differences used in the *t*-tests differ because not every person completed a match for both music and speech.

**Figure 6:**
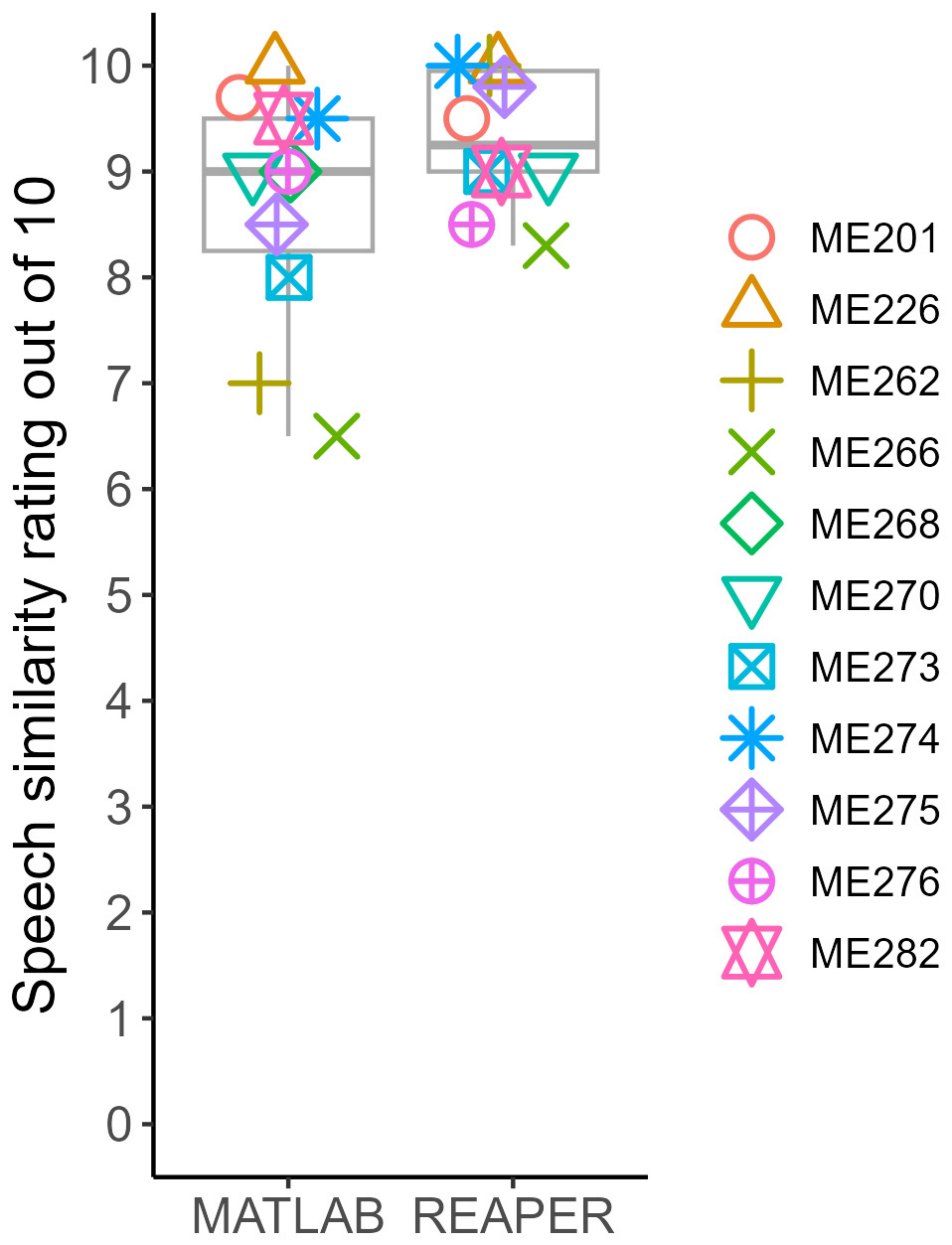
Validation of REAPER approach as compared to previous MATLAB approach for generating speech matches.

Finally, we wanted to validate the REAPER approach as compared to the MATLAB approach as described in Dorman et al. (2020) and reviewed in the Introduction. The scores are shown in Figure 6. A paired-sample *t*-test (not including ME268, who did not complete the REAPER approach for speech) revealed no significant mean difference between the ratings with the new REAPER approach as compared to the MATLAB approach for speech scores, t (9) = 1.78, *p* = 0.11, mean difference 0.64, 95% CI: (−0.17, 1.45). REAPER speech scores were on average 9.3 out of 10 (median: 9.25 out of 10, range: 8.3 to 10) while the MATLAB approach speech scores were on average 8.7 out of 10 (median: 9 out of 10, range: 6.5 to 10).

## 4 Discussion

This exploratory study aimed to characterize the perceptual differences between speech and music sound quality as experienced by SSD-CI listeners and to establish a methodology using commercially available software for generating sound quality matches to both types of signals. We hypothesized that, due to the greater temporal and spectral complexity of music relative to speech, similarity ratings for music would be lower than those achieved for speech, and that more extensive acoustic manipulations would be required to approximate musical sound quality. We found that music similarity scores were, indeed, significantly lower than speech similarity scores. However, the difference in the number of plugins used was not statistically significant. Even with the possibility of using additional plugins, music matches were less similar to the CI percept than speech matches (see Supplementary material for details on speech and music matches). Several participants could not achieve acceptable music matches despite extensive attempts. We were able to successfully generate high similarity ratings to speech and music for both musicians and non-musicians, suggesting that this method of obtaining similarity matches is not limited to participants with musical experience.

For speech, most patients selected bandpass filtering, distortion, and pitch shifting to achieve high similarity ratings, consistent with prior speech-matching work (Dorman et al., 2020, 2024). The similarity in mean match scores for speech using REAPER-based parameters (9.3 out of 10) and using the parameters in Dorman et al. (2020), i.e, 8.7 out of 10, indicates that commercially available software, i.e., REAPER, can be used when assessing the sound quality of speech for SSD-CI listeners. The use of commercially available software will enable other researchers to replicate and expand our study. It would be valuable to establish whether our results can be replicated using other DAWs and audio processing tools.

The specific plugins selected to create matches to speech and music can inform our understanding of factors underlying the sound quality of CIs. The number of plugins needed to achieve a similarity rating varied widely across participants, which could have reflected the wide range of patient demographics, including musical experience, hearing thresholds, and experience with their CI. Many participants used distortion and compression plugins, likely reflecting abnormalities in envelope-based, signal coding (Limb and Roy, 2014). Several participants reported hearing monotonic sounds rather than melodies, consistent with well-documented pitch perception deficits in CI users (Bleckly et al., 2024). The within-participant design used in this study controls for individual differences in both speech and music perception. Future work should explore how device signal processing and mapping parameters relate to the specific plugins required for matching.

A finding from one participant warrants comment. ME266 was implanted at the same time as acoustic neuroma removal and this participant's matches differed qualitatively from others. ME266 emphasized that speech intelligibility was distinct from sound quality—at times, the sound quality was perceptually close, but the participant refused high similarity ratings because intelligibility was too high in the simulated model. Eleven plugins were needed to achieve a speech match due to this intelligibility-quality discrepancy, and we could not achieve a music match because the described timbral transformations could not be generated with available plugins. Acoustic neuroma patients may represent a unique subset of SSD users for future study.

These findings have important clinical implications. Poor sound quality for speech and music has measurable consequences for device use and quality of life. Non-use rates triple from 6% in users with bilateral sensorineural hearing loss (Summerfield and Marshall, 2000) to 18% among SSD users who possess a typical-hearing acoustic comparison ear (Tan et al., 2024), with poor sound quality frequently cited as a primary factor (Neukam et al., 2024). In addition, poor sound quality correlates with reduced daily device wear time (Busch et al., 2017). Moreover, accumulating evidence suggests that sound quality may be a stronger predictor of quality of life than speech recognition performance (McRackan et al., 2018; Berg et al., 2025a). Despite this, current clinical frameworks emphasize speech outcomes almost exclusively while neglecting systematic sound-quality optimization.

The clinical significance of understanding musical sound quality specifically is substantial: 82% of CI users describe music as sounding unnatural, mechanical, or distressing (Philips et al., 2012; Bleckly et al., 2024). CI users can identify musical instruments only about 45% of the time, while normal-hearing listeners achieve 94% accuracy (Kang et al., 2009; Drennan et al., 2015). These outcomes reflect problems in signal resolution that are common to both music and speech. A partial list includes (i) a spectral representation that is both sparse, broad and, in many cases, upshifted in frequency (Wilson, 2015), (ii) frequency resolution on the order of 10 times poorer than normal (Zeng, 2002), (iii) a reduction in the number of discriminable steps in intensity (Nelson et al., 1995), and (iv) an amplitude/time representation that does not faithfully mimic the amplitude envelope of the signal (Wilson et al., 1997). To add to the problem of obtaining a faithful representation of speech and music signals, studies have shown differences in auditory cortex activation for signals delivered by a CI (Sharma et al., 2016). Although the problems in signal resolution listed here alter the sound of both speech and music, our data indicate that the sound of music bears a greater burden, even for familiar music.

### 4.1 Limitations

Several limitations of our work may reduce generalizability. First, the method depends on the moderator-participant relationship. Developing shared language and workflow takes time, and participants may adjust ratings to satisfy the moderator. That said, some patients gave high similarity ratings for speech but not for music. Second, we did not use the same stimuli across matching methods. Nearly all participants listened to “The sun is finally shining,” but not all participants listened to all speech stimuli. Moreover, participants chose different musical passages for testing. Using this approach, there was a bias toward familiar music. Future studies should compare the sound quality of familiar and unfamiliar music. Third, we only used DAI via cable to the CI. Other delivery methods exist, e.g., circumaural headphones, Roger pen and AudioLink streaming. These alternative input systems may color the signal in a different manner than DAI. Fourth, the participant population was heterogeneous and relatively small, including both SSD and bimodal users with varying audiometric profiles in the non-implanted ear. Although this project was a successful demonstration of feasibility, future projects could investigate whether this work generalizes to larger samples of each population. While SSD-CI users (with normal hearing in the ear contralateral to the implant) provide ideal within-person comparisons, participants with partial hearing and a hearing aid in the contralateral ear may attend to different aspects of the signal.

### 4.2 Directions for further research

This methodology provides a foundation for future research on CI sound quality optimization. Fruitful directions include: (1) longitudinal matching from activation through the 1st year to understand how sound quality changes with neural adaptation; (2) test-retest reliability of matches to establish stability over time; (3) relationships between device characteristics (e.g., electrode length and insertion depth), mapping parameters (e.g., stimulation levels), and clinical outcomes (e.g., speech recognition and audiometric thresholds) with sound quality matches; (4) comparisons of stimulus delivery methods, particularly given that Shah et al. (2025) found most CI users do not prefer direct streaming over external speakers for music enjoyment; and (5) direct comparisons of familiar vs. unfamiliar music to disentangle perceptual from cognitive contributions to matching. A more controlled stimulus set, standardized relative to musical complexity and participant experience, would also strengthen future studies. Finally, future work should incorporate measures of both perception and enjoyment, as Wright and Uchanski (2012) demonstrated that these are independent constructs in CI users.

## Author's note

Portions of this work were presented at the 2025 CI CRASH in Minneapolis, MN.

## Data Availability

The original contributions presented in the study are included in the article/Supplementary material, further inquiries can be directed to the corresponding author.
