# Supplementary material for: A digital audio workstation approach for matching the sound quality of speech and music for single-sided deaf patients fit with cochlear implants
Source: Front Neurosci. 2026 Apr 9;20:1798569. doi: 10.3389/fnins.2026.1798569 (PMC13102824; doi:10.3389/fnins.2026.1798569)
Supplement: Supplementary file 1 [file Table_1.docx]

**Supplementary materials**

The following summarizes the matches and manipulations made for individual participants. Details about the plugin selections are available in the supplementary tables. The most common plugin type was equalization, followed by distortion, pitch shifting, dynamic range compression, modulation and smear.

ME201 described sound quality akin to “an AM radio that hadn’t been tuned quite right.” The example song was the singing in “Winchester Cathedral” by The New Vaudeville Band. This motivated the bandpass filter. It was relatively straightforward from this point, only two plugins were needed, Waveshaping Distortion and a bandpass filter. Past this, only adjustments to the plugin parameters were needed for different stimuli. The center frequency of the bandpass filter was between 800 Hz and 1.5 kHz with cutoff frequencies of 100 Hz and 4 kHz.

ME226 selected the words “telephone,” “thin,” “computer-like,” and “muffled” to describe his CI, although “computer-like” was not a common experience for him. The CI was reported as muffled, but not in the sense of missing high frequencies. As with ME201, only Waveshaping Distortion and bandpass filtering was needed for a speech match. The bandpass filter pass band was broad, between 600 Hz and 3 kHz, with cutoff frequencies of 500 Hz and 8 kHz. The music match required more frequency specific equalization than the speech samples.

ME262 was a professional audio engineer and was able to help guide the plugin selection and specification of frequencies to filter. A relatively large number of plugins were used for this participant. This was in part because additional sound quality aspects were requested by the participant. For the speech match, the stimulus was routed to a separate track and was used to gate on or off a sinusoid tuned to 174 Hz. This “bouncing” sine tone was mixed back into the speech sample. For the music match, the sine tone was no longer audible, but instead, at certain portions of the vocal performance, a “wah” effect was perceived. This was reconstructed by routing the speech signal to a separate channel, bandpass filtered to the region surrounding 1.5 kHz, and set to trigger an “auto-wah” (where a dynamic bandpass filter is swept as the envelope of the signal changes). In general, the matches used compression, high and low pass filters to restrict the frequency range to between 100 Hz and 10 kHz, some distortion, and a pitch shift of less than one semi-tone. The “smear” plugin was necessary for a good speech similarity score.

ME266 had an acoustic neuroma and emphasized that sound quality did not match speech intelligibility. The sound quality was described as “putting your mouth next to a tambourine or tinfoil.” By applying a low pass filter with a very low cutoff frequency (346 Hz), the participant reported that intelligibility was similar to his CI, but the sound quality was not like that of a low pass filter. A “scoop” was used to remove frequencies near 1 kHz, which could potentially relate to the surgical removal of the acoustic neuroma during implantation. Eventually, a similarity rating higher than 8/10 was achieved with many plugins not used by most other participants, such as delay, flanger, and limiter. This participant was unable to achieve a music quality match for the piano melody, which was reported to sound like “hitting a trash can cover with a mallet” and like a “racquetball” with a “plop” sound. No similarity match was generated because the participant reported a 0 out of 10 match for the selected plugins.

ME270 was a trumpet player and was able to achieve high similarity matches to speech with distortion, band pass filtering, and pitch shifting. Both a piano melody and chromatic scale were attempted for music matching. The pitches of specific notes in the music samples could not be separated from the sound quality. Different sets of plugins were applied to different frequency ranges.

For ME273, ME274, and ME275, high rating similarity matches were not achieved for any music samples. For ME276, music samples required more modulation (flanger) and bit reduction past the band limiting, distortion and smear applied for the speech signal. Finally, for ME282, while high similarity rating speech matches were quickly reached with bandpass filtering, pitch shifting, and distortion, the musical samples had overwhelming “sizzle” associated with high hats and tambourine. This was mimicked with a bandpass filter at 6 kHz with a very high Q (sharp resonant peak) and recreated the “sizzle.”

In summary, many participants could reach a high similarity match with some combination of band-limiting of frequencies between 100 Hz and 8 kHz, pitch shifts within one semitone, distortion or saturation, and a modulation effect (often smear). Music matches usually required adjusting one or all of these plugins, but also often the introduction of at least one more plugin. A few participants required bespoke combinations of plugins to recreate a particularly salient effect, such as a “wah” effect or a “sizzle” that did not seem to occur with all stimuli or in other participants.

Supplementary Materials Table 1: Plugin and sound effects used in REAPER music sound quality matching.

| Plugin name | Plugin type | Plugin description | Parameter(s) |
| --- | --- | --- | --- |
| AutoWah | Automatic wah-wah | Mimics a “wah-wah” pedal that sweeps a bandpass filter as the intensity of the signal changes. | Position, Resonance, Filter Distortion |
| Bit crush | Bit crusher | Reduces the bit resolution to produce a low fidelity sound quality like an old computer game. | Resolution (bits), Input Gain (dB), Dither, Noise Shaping, Dither Type, Highpass Dither |
| Distortion | Distortion | Clips the signal to sound like a very loud guitar amplifier. | Gain (dB), Hardness, Max Volume (dB) |
| Distortion (Fuzz) | Distortion | Clips the signal to sound like a guitar amplifier with distorted transistors. | Shape, Hard Limit (dB), Wet Mix (dB), Dry Mix (dB) |
| Flanger | Flanger | Adds a second, delayed copy of the signal to itself where the delay changes over time, creating a phasing sound. | Length (ms), Feedback (dB), Wet Mix (dB), Dry Mix (dB), Rate (Hz) |
| Limiter | Compression | Prevents signal from exceeding a given intensity without clipping or distorting. | Threshold (dB), Look Ahead, Attack, Hold (ms), Release (ms), Limit (dB), Reduction |
| ReaComp | Compression | Reduces the peaks in a signal past a given intensity. | Pre-comp (ms), Attack (ms), Release (ms), Ratio, Knee Size (dB), Threshold (dB) |
| ReaDelay | Delay | Adds a second, delayed copy of the signal to itself to reproduce an echo effect. | Length (ms), Feedback (dB), Lowpass Filter (Hz), Highpass Filter (Hz), Resolution (bits), Stereo Width |
| ReaEQ | Equalization | A time-domain reduction or increase in specific frequencies, typically to cut only low or high frequencies. | Frequency (Hz), Gain (dB), Bandwidth (Octaves) |
| ReaFIR | Equalization | A frequency-domain reduction or increase in specific frequencies, typically to achieve an unusual frequency profile. | Points can be added to “draw” the shape of the filter |
| ReaPitch | Pitch shift | Adjusts the pitch or formants in a signal to a lower or higher frequency. | Pitch Shift (semitones), Format Shift (semitones), Wet Mix (dB), Dry Mix (dB) |
| Tone Generator | Sine wave generator | Generates a sine tone at a specific frequency. | Wet Mix (dB), Dry Mix (dB), Base Frequency (Hz), Shape |
| Ring Modulator | Ring modulator | Mimics ring modulation, where two unrelated signals are combined to create a new signal that may contain non-harmonic information. | Mod Frequency, Feedback (%), Non-Linearities (%), Mix (%), Output (dB) |
| Saturation | Distortion | Clips the signal with a sigmoidal mapping to create “soft” distortion. | Amount (%) |
| Smear | Temporal smear | Custom plugin to mimic smearing experienced by people with hearing loss and broadened auditory filterbanks (Baer & Moore, 1994). | FFT Size, Cutoff Frequency (Hz), Overlap Factor, Alpha Controlling Spread |
| (STFT) Short-Time Fourier Transform | Low-pass filter | Custom plugin with a very sharp low pass filter cutoff. | FFT Size, Cutoff Frequency (Hz) |
| White Noise Generator | White noise generator | Generates white noise. | Dry Volume (dB), Noise Volume (dB) |
| WS Distortion | Distortion | Clips the signal with an aggressive sigmoidal mapping to create somewhat “soft” distortion. | Distortion (%) |

Supplementary Materials Table 2: List of quality descriptors from Dorman et al. (2025) for speech samples.

| Quality Descriptors |
| --- |
| Boomy |
| Clear |
| Computer-like |
| Distorted |
| Far-away |
| Full |
| Grainy |
| Grungy |
| Harsh |
| High-pitched |
| Hollow |
| Metallic |
| Mickey Mouse-like |
| Muddy |
| Muffled |
| Nasal |
| Rich |
| Reverberant |
| Shrill |
| Smeared |
| Smooth |
| Telephone-like |
| Thin |
| Treble-y |
| Warm |
| Please provide another adjective that is not on this list, if you feel it is more appropriate to describe what you are hearing. |

Supplementary Materials Table 3: Full list of plugins or effects used to achieve each match.

| ME201 | | | | |
| --- | --- | --- | --- | --- |
| **REAPER - Speech** | | | | |
| *Stimulus* | *Number of plugins* | *Match descriptors* | *Missing?* | *Rating out of 10* |
| The sun is finally shining | 2 plugins | WS Distortion 89.7%, ReaFIR 100-3.5 kHz pass band cf 800 Hz |  | 9 |
| The boy enjoyed dancing with his dog | 2 plugins | WS Distortion 75.2%, ReaFIR 100-3.5 kHz pass band cf 800 Hz |  | 9 |
| I wish she would convince herself she could fly | 2 plugins | WS Distortion 63.3%, ReaFIR 100-3.5 kHz pass band cf 800 Hz | Needed tuning | 9 |
| I wish she would convince herself she could fly | 2 plugins | WS Distortion 63.3%, ReaFIR 100-4 kHz pass band cf 1.4 kHz |  | 9.5 |
| **REAPER - Music** | | | | |
| *Stimulus* | *Number of plugins* | *Match descriptors* | *Missing?* | *Rating out of 10* |
| Piano melody (highest register) | 3 plugins | PitchShift -1 semitones, Distortion 17 shape -12 dB hard limit -7 dB wet mix -120 dB dry mix, ReaFIR 100-4 kHz peak in pass band 1.4 kHz |  | 8.5 |
| Piano melody (lower register) | 3 plugins | PitchShift -1 semitones, Distortion 17 shape -12 dB hard limit -7 dB wet mix -120 dB dry mix, ReaFIR 100-4 kHz pass band cf 1.4 kHz | Difficulty hearing attack and sustain | 9 |
| Country Roads | 2 plugins | WS Distortion 91.2%, ReaFIR 100-4 kHz pass band cf 1.4 kHz |  | 9 |
| Postcard to Paris | 2 plugins | WS Distortion 91.2%, ReaFIR 100-4 kHz pass band cf 1.4 kHz |  | 9 |
| Perfect Symphony (Sheeran singing) | 2 plugins | WS Distortion 91.2%, ReaFIR 100-4 kHz pass band cf 1.4 kHz | Voice is not clean enough, depending on whether there were other instruments | 9.5 |
| Perfect Symphony (Bocelli singing) | 2 plugins | WS Distortion 80.8%, ReaFIR 100-4 kHz pass band cf 1.4 kHz |  | 9.5 |
| **MATLAB - Speech** | | | | |
| *Stimulus* | *Number of plugins* | *Match descriptors* | *Missing?* | *Rating out of 10* |
| The sun is finally shining | 3 effects | Pitch +35 Hz, Metallic .996, Low pass filter 2000 Hz | It is hard to describe. Perhaps pitch is not quite right. | 9.7 |

| ME226 | | | | |
| --- | --- | --- | --- | --- |
| **REAPER - Speech** | | | | |
| *Stimulus* | *Number of plugins* | *Match descriptors* | *Missing?* | *Rating out of 10* |
| The sun is finally shining | Clinical  2 plugins | WS Distortion 59.7%, ReaFIR 300-8500 Hz pass band |  | 10 |
| The boy enjoyed dancing with his dog | 2 plugins | WS Distortion 59.7%, ReaFIR 300-5000 Hz pass band |  | 10 |
| I wish she would convince herself she could fly | 2 plugins | WS Distortion 59.7%, ReaFIR 300-5000 Hz pass band |  | 10 |
| **REAPER - Music** | | | | |
| *Stimulus* | *Number of plugins* | *Match descriptors* | *Missing?* | *Rating out of 10* |
| Hello, Goodbye | 4 plugins | Distortion 20 dB gain 6 hardness -12 max volume, ReaFIR 700-3000 Hz passband, ReaEQ notch -3.8 dB @ 2177 Hz with 0.62 oct bw, ReaEQ notch -9.4 dB @ 3737 Hz with 0.12 oct bw | Vocals still need to be less clear and have more sibilance, to get 10/10, drums would need to be more clear, vocals need to be less intelligible and have harsh sibilance | 9 |
| Sympathy for the Devil | 2 plugins | Distortion (Fuzz) 11 shape 1 dB hard limit -12 dB wet mix -24 dry mix, ReaFIR 700-3000 Hz passband |  | 9 |
| **MATLAB - Speech** | | | | |
| *Stimulus* | *Number of plugins* | *Match descriptors* | *Missing?* | *Rating out of 10* |
| The sun is finally shining | 3 effects | Smear 3, LP 1500, HP 400 | Nothing | 10 |

| ME262 | | | | |
| --- | --- | --- | --- | --- |
| **REAPER** **-** **Speech** | | | | |
| *Stimulus* | *Number of plugins* | *Match descriptors* | *Missing?* | *Rating out of 10* |
| The sun is finally shining | 10 plugins | ReaEQ low pass 13000 Hz with 2 oct bw, ReaEQ high pass 82 Hz with 2 oct bw, ReaPitch -1.5 semitones, Smear 256 FFT size 7555 Hz cutoff 18 overlap factor 0.2 spread, Distortion 1 dB gain 1 hardness -28 dB max volume, ReaFIR 9 dB boost @ 120 Hz and -6 dB cut @ 400 Hz and -3 dB cut at 800 Hz, STFT 4096 FFT size 20000 Hz cutoff, Tone generator 174 Hz , ReaGate @ -11.9 dB 3 ms attack 202 ms release, ReaComp -32.8 dB threshold inf ratio 3 ms attack 100 ms release |  | 10 |
| **REAPER - Music** | | | | |
| *Stimulus* | *Number of plugins* | *Match descriptors* | *Missing?* | *Rating out of 10* |
| Mysterious Ways | 10 plugins | ReaComp -26 dB threshold 100 ms attack 400 ms release 3:1 ratio, ReaComp -17 dB threshold 4 ms attack 16 ms release 3:1 ratio, ReaEQ low pass 10000 Hz with 2 oct bw, ReaEQ high pass 82 Hz with 2 oct bw with -4 dB notch at 2800 Hz, ReaPitch -1.5 semitones, Smear 256 FFT size 7555 Hz cutoff 18 overlap factor 0.2 spread, WS 93.3%, ReaFIR 9 dB boost @ 120 Hz and -3 dB cut at 800 Hz, STFT 4096 FFT size 20000 Hz cutoff, Master Limiter -10 dB threshold 85 ms look ahead 100 ms attack 400 ms release -6 dB limit | “Watery” quality that depended on the song, and sounded like a moving envelope sweep or “wah wah” | 8 |
| Mysterious Ways | 16 plugins | Same as above except with STFT cutoff at 6500 Hz, and copy of signal routed to ReaEQ bandpass 1146 Hz peak with -120 dB gain and 0.82 oct bw, ReaGate -26 dB threshold 3 ms attack 100 ms release, Wah-wah triggered by signal with 0.93 top resonance 0.66 bottom resonance and 0 filter distortion, ReaPitch down an octave 9.41 increase in formant semitones, ReaComp -36 dB threshold 43 ms attack 172 ms release 10:1 ratio, ReaFIR high pass with cutoff of 120 Hz |  | 9 |
| **MATLAB - Speech** | | | | |
| *Stimulus* | *Number of plugins* | *Match descriptors* | *Missing?* | *Rating out of 10* |
| The sun is finally shining | 3 effects | Smear 5, Low pass 10000 Hz, High pass 80 | The real difference is that the mid tones in the middle is dominant in the CI. Boost 350-700 Hz | 7 |

| ME266 | | | | |
| --- | --- | --- | --- | --- |
| **REAPER - Speech** | | | | |
| *Stimulus* | *Number of plugins* | *Match descriptors* | *Missing?* | *Rating out of 10* |
| The sun is finally shining | 6 plugins | ReaEQ low pass 1020 Hz with 0.2 dB gain 0.74 oct bw, ReaEQ high pass 758 Hz with 0 dB gain and 2 oct bw, ReaPitch -11 cents +1 semitones, Distortion (Fuzz), 108 shape -12 dB hard limit -7 dB wet mix 0 dB dry mix, ReaFIR -24 dB notch at 900 Hz, STFT low pass at 800 Hz 4096 FFT size |  | 8 |
| The boy enjoyed dancing with his dog | 10 plugins | ReaComp -17 dB threshold 4 ms attack 16 ms release 3:1 ratio, ReaEQ low pass 1020 Hz with 0.2 dB gain 0.74 oct bw, ReaEQ high pass 758 Hz with 0 dB gain and 2 oct bw, ReaPitch --11 cents -1 semitones, Flanger, Distortion (Fuzz), 108 shape -12 dB hard limit -7 dB wet mix 0 dB dry mix, ReaFIR -24 dB notch at 900 Hz, ReaDelay 4,3 ms delay, STFT low pass at 800 Hz 4096 FFT size, Master limiter -13.6 dB threshold 85 ms look head 100 ms attack 400 ms release -6 dB limit | CI sounded more metallic/robotic than the acoustic ear, without actually sounding like the effect of either of those descriptors | 8.3 |
| **REAPER - Music** | | | | |
| *Stimulus* | *Number of plugins* | *Match descriptors* | *Missing?* | *Rating out of 10* |
| Piano melody |  | Could not complete match |  | 0 |
| Uncle John’s Band |  | Could not complete match |  | 0 |
| **MATLAB - Speech** | | | | |
| *Stimulus* | *Number of plugins* | *Match descriptors* | *Missing?* | *Rating out of 10* |
| The sun is finally shining | 4 effects | Smear 10, Metallic .999, Low pass filter 1500 Hz, High pass 400 Hz | Need to find a way to make the midranges lower and add some 'sss' to s and th sounds. Instead of getting shining I get 'shinin'​  The distortion in the simulation is consistent across the sentence where it goes up and down in the CI ear.  I'm getting more at the beginning and ending of words. Very similar issues to the other two maps so far. | 6.5 |

| ME268 | | | | |
| --- | --- | --- | --- | --- |
| **REAPER - Speech** | | | | |
| *Stimulus* | *Number of plugins* | *Match descriptors* | *Missing?* | *Rating out of 10* |
| Hey Jude | 4 plugins | ReaPitch -2 semitones, WS Distortion 80.3%, ReaFIR 250-3000 Hz passband, ReaDelay 0.4 ms 8065 Hz lpf on delay -4.8 wet mix |  | 8 |
| Bohemian Rhapsody | 4 plugins | ReaPitch -2 semitones, WS Distortion 80.3%, ReaFIR 250-3000 Hz passband, ReaDelay 0.4 ms 8065 Hz lpf on delay -4.8 wet mix |  | 5 |
| Over the Rainbow | 6 plugins | ReaEQ bandpass @ 3030 Hz 0 dB gain 0.32 oct bw, Distortion 9 dB gain 3 hardness -39 dB max volume, ReaPitch -2 semitones, WS Distortion 80.3%, ReaFIR 250-3000 Hz passband, ReaDelay 0.4 ms 8065 Hz lpf on delay -4.8 wet mix |  | 9 |
| Bohemian Rhapsody | 6 plugins | ReaEQ bandpass @ 3030 Hz 0 dB gain 0.32 oct bw, Distortion 9 dB gain 3 hardness -39 dB max volume, ReaPitch -2 semitones, WS Distortion 80.3%, ReaFIR 250-3000 Hz passband, ReaDelay 0.4 ms 8065 Hz lpf on delay -4.8 wet mix |  | 7 |
| **REAPER - Music** | | | | |
| *Stimulus* | *Number of plugins* | *Match descriptors* | *Missing?* | *Rating out of 10* |
| The sun is finally shining | 4 effects | Pitch -10, Smear 3, Metallic .996, LP 3000 Hz | It's like there is a slight low frequency reverb or something on the CI side | 9 |
| **MATLAB - Speech** |  |  |  |  |
| *Stimulus* | *Number of plugins* | *Match descriptors* | *Missing?* | *Rating out of 10* |
| The sun is finally shining | 4 effects | Pitch -10, Smear 3, Metallic .996, LP 3000 Hz | It's like there is a slight low frequency reverb or something on the CI side | 9 |

| ME270 | | | | |
| --- | --- | --- | --- | --- |
| **REAPER - Speech** | | | | |
| *Stimulus* | *Number of plugins* | *Match descriptors* | *Missing?* | *Rating out of 10* |
| The sun is finally shining | 4 plugins | ReaPitch -2 semitones +1 formant shift, WS distortion 95.4, ReaFIR with 800 Hz peak rolled off to -12 dB at 50 Hz and -3 dB by 4000 Hz, STFT 4096 FFT size 4580 Hz low pass cutoff |  | 9 |
| The sun is finally shining | 5 plugins | ReaPitch -2 semitones +1 formant shift, WS distortion 95.4, ReaFIR with 800 Hz peak rolled off to -12 dB at 50 Hz and -3 dB by 4000 Hz, STFT 4096 FFT size 6700 Hz low pass cutoff, ReaFIR passband peak at 500 Hz -25 dB down at 100 Hz and -18 dB down at 3000 Hz |  | 9 |
| **REAPER - Music** | | | | |
| *Stimulus* | *Number of plugins* | *Match descriptors* | *Missing?* | *Rating out of 10* |
| Piano melody |  | Could not complete |  |  |
| Chromatic scale | 12 plugins | ReaPitch -2 semitones +1 formant shift, WS distortion 95.4, ReaFIR with 800 Hz peak rolled off to -12 dB at 50 Hz and -3 dB by 4000 Hz, STFT 4096 FFT size 6700 Hz low pass cutoff, ReaFIR passband peak at 500 Hz -25 dB down at 100 Hz and -18 dB down at 3000 Hz, routed to 3 instances of ReaEQ with cutoffs of 200 Hz, 200 to 1000 Hz, and 1000 Hz (low, middle, high bands), ReaGate, RingMod, and ReaEQ applied to middle band, RindMod applied to high band |  | 5 |
| **MATLAB - Speech** | | | | |
| *Stimulus* | *Number of plugins* | *Match descriptors* | *Missing?* | *Rating out of 10* |
| The sun is finally shining | 4 effects | Pitch -15 Hz, Smear 10, LP 4000 Hz, HP 300 Hz | The nuanced difference between them are so small that it is very close. But it is still too clear. The texture of the sound is a very good/tight match. She can just hear it too well. More of a fullness to the lower frequencies if we could have an EQ for the lows and turn them up. It's not a distortion or muffle it's just the presence of those lower frequencies is more prominent. | 9 |

| ME273 | | | | |
| --- | --- | --- | --- | --- |
| **REAPER - Speech** | | | | |
| *Stimulus* | *Number of plugins* | *Match descriptors* | *Missing?* | *Rating out of 10* |
| The sun is finally shining | 6 plugins | ReaComp -26 dB threshold 100 ms attack 400 ms release 3:1 ratio, ReaComp -17 dB threshold 4 ms attack 16 ms release 3:1 ratio, Formant shift Smear 256 FFT size 6300 Hz cutoff 10 overlap 0.3 alpha spread, RingMod 40 mod freq 3.6% feedback 22% mix 10 % non-linearities, STFT 4096 FFT size 1186 Hz cutoff | Garble quality is different in CI ear, more garbled, garble is the main quality, density is missing | 9 |
| The sun is finally shining |  |  |  | 10 |
| **REAPER - Music** | | | | |
| *Stimulus* | *Number of plugins* | *Match descriptors* | *Missing?* | *Rating out of 10* |
| Piano melody | 7 plugins | As above but with ReaFIR with pass band between 250 and 500 Hz | Sounds like muffled bass drum, almost like instrument is being replaced | 3 |
| **MATLAB - Speech** |  |  |  |  |
| Stimulus | Number of plugins | Match descriptors | Missing? | Rating out of 10 |
| The sun is finally shining | 3 plugins | Pitch 20, smear 5, 1850 LP | Hearing ear is less garbled than CI | 8 |

| ME274 | | | | |
| --- | --- | --- | --- | --- |
| **REAPER - Speech** | | | | |
| *Stimulus* | *Number of plugins* | *Match descriptors* | *Missing?* | *Rating out of 10* |
| The sun is finally shining | 2 plugins | Metallic, smear |  | 10 |
| **MATLAB - Speech** |  |  |  |  |
| *Stimulus* | *Number of plugins* | *Match descriptors* | *Missing?* | *Rating out of 10* |
| The sun is finally shining | 1 effect | Smear 3 | It almost sounds like the normal ear. | 9.5 |

| ME275 | | | | |
| --- | --- | --- | --- | --- |
| **REAPER - Speech** | | | | |
| *Stimulus* | *Number of plugins* | *Match descriptors* | *Missing?* | *Rating out of 10* |
| The sun is finally shining | 1 plugin | WS distortion 86% |  | 9.8 |
| **REAPER - Music** |  |  |  |  |
| *Stimulus* | *Number of plugins* | *Match descriptors* | *Missing?* | *Rating out of 10* |
| Music from a Sushi Restaurant | 2 plugins | WS distortion 73.9%, ReaFIR pass band 150 to 10000 Hz, -3 dB dip at 1200 Hz | CI side is muddier, acoustic ear is too clear | 6.5 |
| **MATLAB - Speech** |  |  |  |  |
| *Stimulus* | *Number of plugins* | *Match descriptors* | *Missing?* | *Rating out of 10* |
| The sun is finally shining | 3 plugins | Pitch 10 Hz, Smear 6, HP 400 Hz | CI feels mechanical but not robotic. Same crispness but CI seems a little mechanical. ​ | 8.5 |

| ME276 | | | | |
| --- | --- | --- | --- | --- |
| **REAPER - Speech** | | | | |
| *Stimulus* | *Number of plugins* | *Match descriptors* | *Missing?* | *Rating out of 10* |
| The sun is finally shining | 9 plugins | ReaEQ high pass cutoff 900 Hz 0 dB gain 2 oct bw, Smear 256 FFT size 7500 Hz cutoff 16 overlap 0.2 alpha spread, WS Distortion 95%, STFT 4096 FFT 4000 Hz cutoff frequency, routed to ReaComp -42 dB threshold 2 ms attack 100 ms release inf ratio, then routed to White noise generator, ReaEQ low pass 10000 Hz, ReaEQ high pass 930 Hz, Distortion 14 dB gain 1 hardness -22 dB max volume |  | 8 |
| The sun is finally shining | 10 plugins | Above with ReaFIR bandstop filter at -10 dB around 3000 Hz passband below 300 Hz and above 6000 Hz added before STFT in main signal chain |  | 8.5 |
| **REAPER - Speech** | | | | |
| *Stimulus* | *Number of plugins* | *Match descriptors* | *Missing?* | *Rating out of 10* |
| Bob Caygeon | 7 plugins | ReaEQ high pass cutoff 900 Hz 0 dB gain 2 oct bw, Smear 256 FFT size 7500 Hz cutoff 16 overlap 0.8 alpha spread, Flanger 2 ms -120 dB feedback -5 dB wet -6 dB dary 0.4 Hz rate, WS Distortion 95%, ReaFIR bandstop filter at -10 dB around 3000 Hz passband below 300 Hz and above 6000 Hz, Bit reduction 4 bits, STFT 4096 FFT 4000 Hz cutoff frequency |  | 8 |
| **MATLAB - Speech** | | | | |
| *Stimulus* | *Number of plugins* | *Match descriptors* | *Missing?* | *Rating out of 10* |
| The sun is finally shining | 4 effects | Pitch 20 Hz, Formant shift 50 Hz, Smear 2, High pass 600 Hz | A little more robotic but I can't quite describe it. Maybe thinner. The difference between an AI voice and a normal voice. | 9 |

| ME282 | | | | | |
| --- | --- | --- | --- | --- | --- |
| **REAPER - Speech** | | | | | |
| *Stimulus* | *Number of plugins* | *Match descriptors* | | *Missing?* | *Rating out of 10* |
| The sun is finally shining | 3 plugins | ReaPitch +1 semitone, WS distortion 77%, ReaFIR passband between 400 Hz and 2000 Hz | |  | 9 |
| The boy enjoyed dancing with his dog | 5 plugins | ReaComp -26 dB threshold 100 ms attack 400 ms release 3:1 ratio, ReaComp -17 dB threshold 4 ms attack 16 ms release 3:1 ratio, ReaPitch -2 semitone, WS distortion 62.4%, ReaFIR passband between 200 Hz and 7000 Hz | |  | 8.4 |
| **REAPER - Music** | | | | | |
| *Stimulus* | *Number of plugins* | *Match descriptors* | | *Missing?* | *Rating out of 10* |
| Don’t Stop | 4 plugins | ReaComp -26 dB threshold 100 ms attack 400 ms release 3:1 ratio, ReaComp -17 dB threshold 4 ms attack 16 ms release 3:1 ratio, WS distortion 62.4%, ReaFIR passband between 500 Hz and 2000 Hz with +24 peak added at 6000 Hz for sizzle | |  | 8 |
| Uncle John’s Band | 4 plugins | ReaComp -26 dB threshold 100 ms attack 400 ms release 3:1 ratio, ReaComp -17 dB threshold 4 ms attack 16 ms release 3:1 ratio, WS distortion 75%, ReaFIR passband between 500 Hz and 2000 Hz with +24 peak added at 6000 Hz for sizzle | | Sounds like back in the day trying to match the AM radio dial | 8.5 |
| Thriller | 4 plugins | ReaComp -26 dB threshold 100 ms attack 400 ms release 3:1 ratio, ReaComp -17 dB threshold 4 ms attack 16 ms release 3:1 ratio, WS distortion 62.4, ReaFIR passband between 500 Hz and 2000 Hz with +24 peak added at 6000 Hz for sizzle | | Harder to get close | 8 |
| **MATLAB - Speech** | | | | | |
| *Stimulus* | *Number of plugins* | *Match descriptors* | *Missing?* | | *Rating out of 10* |
| The sun is finally shining | 1 effect | HP 2000 Hz | The CI ear feels more direct verses the ear feels like it's coming from outside of my head through my ear. The CI thus feels little bit more intense on the syllables. | | 9.5 |
